# Aligned contiguous microfiber platform enhances neural differentiation of embryonic stem cells

**DOI:** 10.1038/s41598-018-24522-9

**Published:** 2018-04-17

**Authors:** Zhenjie Liu, Zhengqing Hu

**Affiliations:** Department of Otolaryngology-HNS Wayne State University School of Medicine Detroit, Michigan, 48201 USA

## Abstract

A microfiber platform that is able to enhance neuronal differentiation and guide aligned neurite outgrowths is essential to the repair of nerve damage. To achieve this aim, we utilized biocompatible and biodegradable poly lactic-co-glycolic acid (PLGA) to design a novel Aligned Contiguous Microfiber Platform (ACMFP) as substrates for the neuronal induction of mouse embryonic stem (ES) cells. To generate the ACMFP, a modified micro-fluid chip system was established to control microfiber parameters including fiber diameter, alignment, and the distance between fibers. Further, Pluronic-F127 was applied to the ACMFP system to maintain a stable and highly aligned fiber platform for at least 12 days. We found that the ACMFP can enhance the neuronal differentiation of mouse ES cells. The ACMFP system showed significantly better neurite outgrowth alignment guidance compared to the control substrate. The effects of alignment guidance were inversely proportionate to the diameter of the fiber, with the optimal diameter size of 60 µm. This study demonstrates a novel ACMFP system that can be used as a biomaterial substrate for neurite outgrowth alignment guidance, which may provide a new model for the development of a multidisciplinary treatment option for nerve injuries.

## Introduction

Nerves that connect the brain and the rest of the body can be damaged by overpressure, stretch, contusion, laceration or other neurodegenerative diseases^1–3^. Mild injuries to nerve are usually repaired automatically with minutes or for several weeks, whereas a surgery and/or biological nerve replacement is needed for severe nerve injuries involving disrupted or broken nerve fibers^4,5^. Since embryonic stem (ES) cells are pluripotent cells that are able to differentiate into all types of cells of the body including neurons with their nerve fibers, they have been suggested for the replacement therapy for nerve injuries^6–11^. ES cell-derived neurons that are cultured on the culture dish substrates often demonstrate neurite growth in random orientations^12,13^. However, aligned nerve fibers are usually essential for proper nerve functions. Therefore, how to guide aligned nerve fiber growth is a critical issue for a successful stem cell-based nerve replacement treatment.

Biomaterial products with either nano- or micro-meter substrate have been suggested to guide neuronal differentiation and/or neurite outgrowth of ES cells^12–15^. A suitable biomaterial is essential for biomaterial substrate generation. Many materials have been used for biomaterial substrate research, including natural polymers chitosan, collagen, alginate, as well as several synthetic biodegradable polymers^16–19^. An ideal biomaterial for the neuronal induction of ES cells for nerve replacement is expected to be biocompatible and biodegradable, without toxicity to tissues/cells and with the capability to degrade upon completion of nerve healing^20,21^. Poly lactic-co-glycolic acid (PLGA) is a biocompatible and biodegradable synthetic material that has been tested in numerous studies^22,23^. PLGA does not show toxicity or cause inflammatory responses *in vitro* or in *vivo*^24–26^. To test its biodegradation, 75:25 PLGA was implanted into animals and it was found that PLGA was fully degraded 8–10 weeks after implantation^27,28^. PLGA possesses the feature of plasticity, which can be produced as fibers, spheres and membranes of different size^15,29–31^. Moreover, PLGA has been approved by Food and Drug Administration (FDA) for clinical applications due to its biocompatibility and biodegradability^22,23^. Because of these features, PLGA was selected for the biomaterial substrate production in this research.

**Figure 1:**
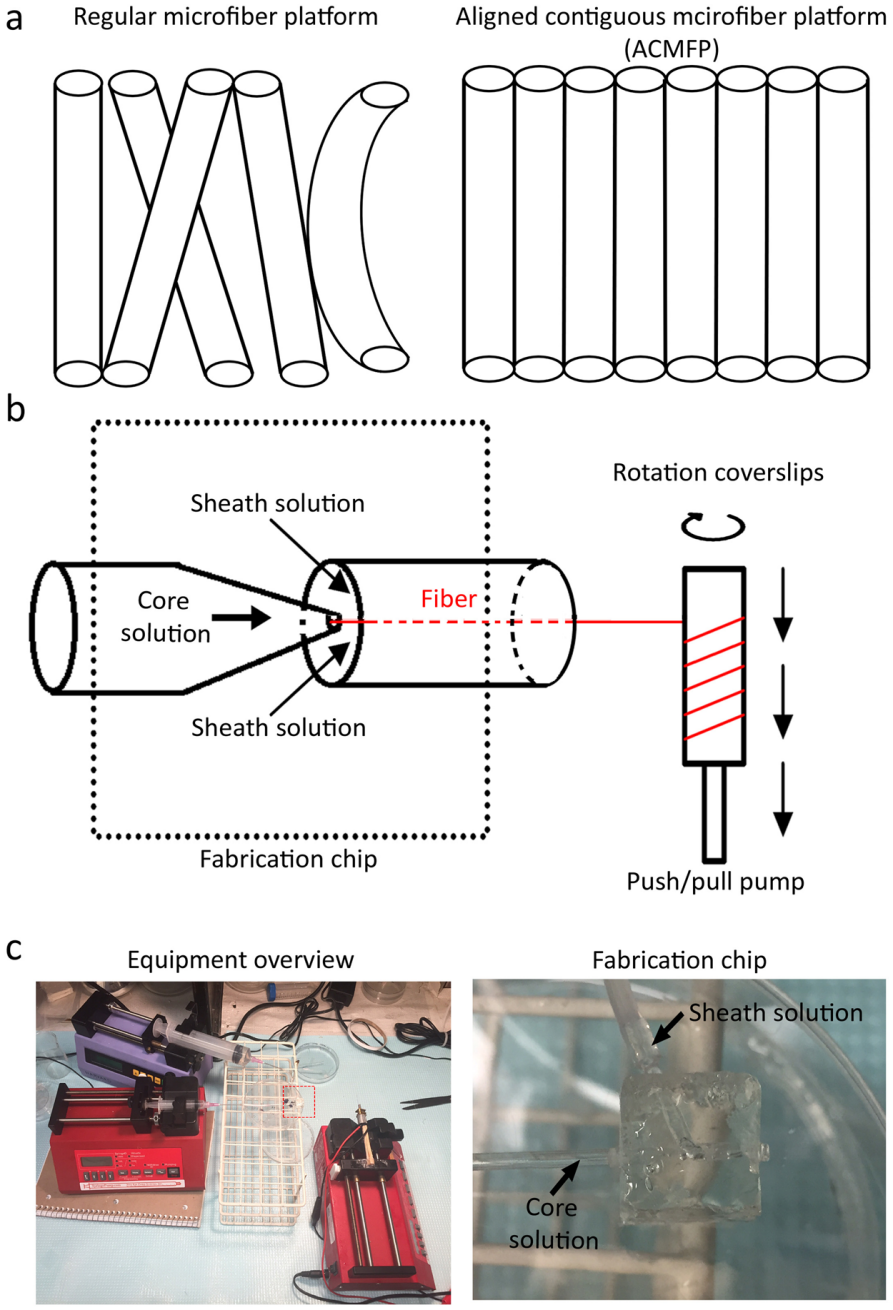
Design and production of the aligned contiguous fiber platform (ACMFP). (**a**) Diagram of regular microfiber platform and aligned contiguous microfiber platform (ACMFP). Regular microfiber platform shows fiber overlap and null space, whereas ACMFP shows a good alignment pattern without overlap or null space. (**b**) Diagram of the ACMFP equipment. The equipment includes two parts, the fabrication chip part, and the collection part. The sheath solution and core solution meet in the chip area to form PLGA fiber. The push/pull pump collects microfibers at a constant speed to prevent fiber overlap. (**c**) Images of the equipment overview and the fabrication chip area.

It is known that nanofibers are able to stimulate neuronal differentiation of ES cells^14^. Due to the electrospinning technology involved in the production of nanofibers, these nanofibers are not strictly parallel, and may have deviations as great as 90^o^ between these fibers^32,33^. Accordingly, the alignment of neurite outgrowths/axons on nanofibers is suboptimal, which may largely limit the function of nerve fibers. Neurite outgrowths have shown relatively parallel nerve fiber growths on submicron- and microfibers^34,35^. However, it remains controversial whether microfibers are able to stimulate the neuronal differentiation of ES cells, which may affect its application in stem cell-based nerve replacement. Additionally, current microfiber technology lacks an efficient collection unit, which results in the production of fibers with remarkable overlap and gap among them^35^ (Fig. 1a). These gaps may cause several weaknesses. First, many cells fall into gaps without attachment to fibers, which may decrease the efficiency of ES cell attachment and differentiation. Second, microfiber alignment is compromised due to these gaps, which subsequently affects nerve fiber alignment. Third, these gaps compose “null space” that is not related to the fiber function, which may influence the overall performance of the biomaterial. To address these issues, we aimed to design a microfiber system to produce a novel Aligned Contiguous Microfiber Platform (ACMFP) for the neuronal differentiation of ES cells and guidance of nerve fibers (Fig. 1a). The advantage of this system is that fibers are highly parallel and adhere to each other with no or very limited gaps. We will study whether this ACMFP is able to affect the neuronal differentiation of ES cells and subsequent neurite outgrowths of ES cell-derived neurons.

## Result

### Fabrication of ACMFP and prevention of fiber shrinkage

**Figure 2:**
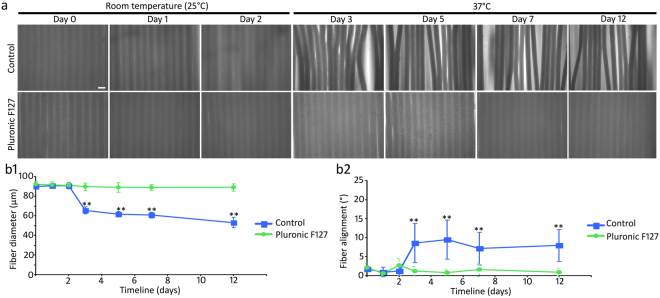
ACMFP shrinkage evaluation and Pluronic F127 treatment. (**a**) Phase contrast microscopy shows 90 μm ACMFPs that are fabricated at room temperature (25 °C), sterilized and maintained at 37 °C for 12 days with or without 40% Pluronic F127 treatment. Day 0: ACMFP production. Day 1: Pluronic F127 or 0.01 M PBS (control) treatment. Day 2: Distilled water rinse to remove residual F127 and 70% alcohol treatment for 15 min to sterilize the fibers. Day 3 to Day 12: ACMFPs in a 37 °C incubator. (**b1**) The diameter of 90 μm ACMFP is evaluated by the ImageJ software from day 0–12 in the control and Pluronic groups (mean ± standard deviation shown in the figure, n = 10 samples per group; **indicates P < 0.01; ANOVA followed by Tukey post hoc test, analysis results shown in Supplemental Table S1). (**b2**) The alignment of 90 μm ACMFP is evaluated by the ImageJ software from day 0–12 in the control and Pluronic groups (mean ± standard deviation shown in the figure, n = 10 samples per group; **indicates P < 0.01; ANOVA followed by Tukey post hoc test, analysis results shown in Supplemental Table S1). Scar bar: 100 μm.

A modified micro-fluid chip fabrication system was established in this research, in which the speed of PLGA and glycerin micro-fluid was controlled by syringe pumps (Fig. 1b,c). When the pump speed of PLGA solution was set at 20 μl/min and glycerin solution at 500 μl/min, 90 µm diameter ACMFP fibers were obtained (90.23 ± 1.75 µm; mean ± standard deviation; Fig. 2a). After a rinse with distilled water for 1 day followed by 70% ethanol incubation for 15 min and distilled water rinse for another day at room temperature, the diameter of fibers was 90.58 ± 3.27 μm (mean ± standard deviation). The diameter of ACMFP fibers was not significantly changed when they were kept in phosphate-buffered saline (PBS) at room temperature (25 °C) for 10–12 days, suggesting that distilled water, PBS and 70% alcohol treatment did not cause obvious fiber diameter change or shrinkage. Next, we test whether temperature affects the diameter by maintaining 90 μm ACMFP fibers in a 37 °C incubator, the body temperature of mammals. It was observed that the diameter of fibers decreased to 65.39 ± 3.62 μm, 61.46 ± 2.78 μm, 60.61 ± 3.01 μm and 53.05 ± 5.23 μm after storage in a 37 °C incubator for 1, 3, 5 and 10 days respectively (mean ± standard deviation; Fig. 2b1). Statistical analysis revealed significant changes (P < 0.01, ANOVA-Tukey test, Supplemental Table S1), suggesting that fibers were observed to shrink when kept at 37 °C. The shrinkage occurred primarily during the first day at 37 °C, and the trend of fiber diameter change slowed down afterward. Variations in fiber diameter seemed to negatively affect fiber alignment. The fiber alignment value deviated from 1.76 ± 0.76° to 8.539 ± 5.19°, 9.46 ± 5.14°, 7.12 ± 4.30° and 7.92 ± 4.21° at 1, 3, 5 and 10 days at 37 °C (mean ± standard deviation; Fig. 2b2), which was statistically significant (P < 0.01, ANOVA-Tukey test, Supplemental Table S1). To prevent fiber shrinkage misalignment, Pluronic F127 was used during fiber fabrication (Fig. 2a). The diameter of fiber in the Pluronic F127 group changed from 92.33 ± 2.39 μm to 89.61 ± 3.54, 88.97 ± 3.02, and 89.93 ± 3.62 μm at day 0, 3, 5, 7 and 12 respectively (mean ± standard deviation; Fig. 2b1), which was not significant (P > 0.05, ANOVA-Tukey test, Supplemental Table S1). The fiber alignment values changed from 2.07 ± 1.33° to 1.25 ± 1.18°, 0.82 ± 0.72°, 1.56 ± 1.74° and 0.95 ± 0.94° at day 0, 3, 5, 7 and 12 respectively (mean ± standard deviation; Fig. 2b2), which was statistically insignificant (P > 0.05, ANOVA-Tukey test, Supplemental Table S1). These results indicate that the fiber diameter and alignment of ACMFP fibers can be maintained by the treatment of Pluronic F127.

### Generation of ACMFP with different fiber size and biocompatibility test of ACMFP

**Figure 3:**
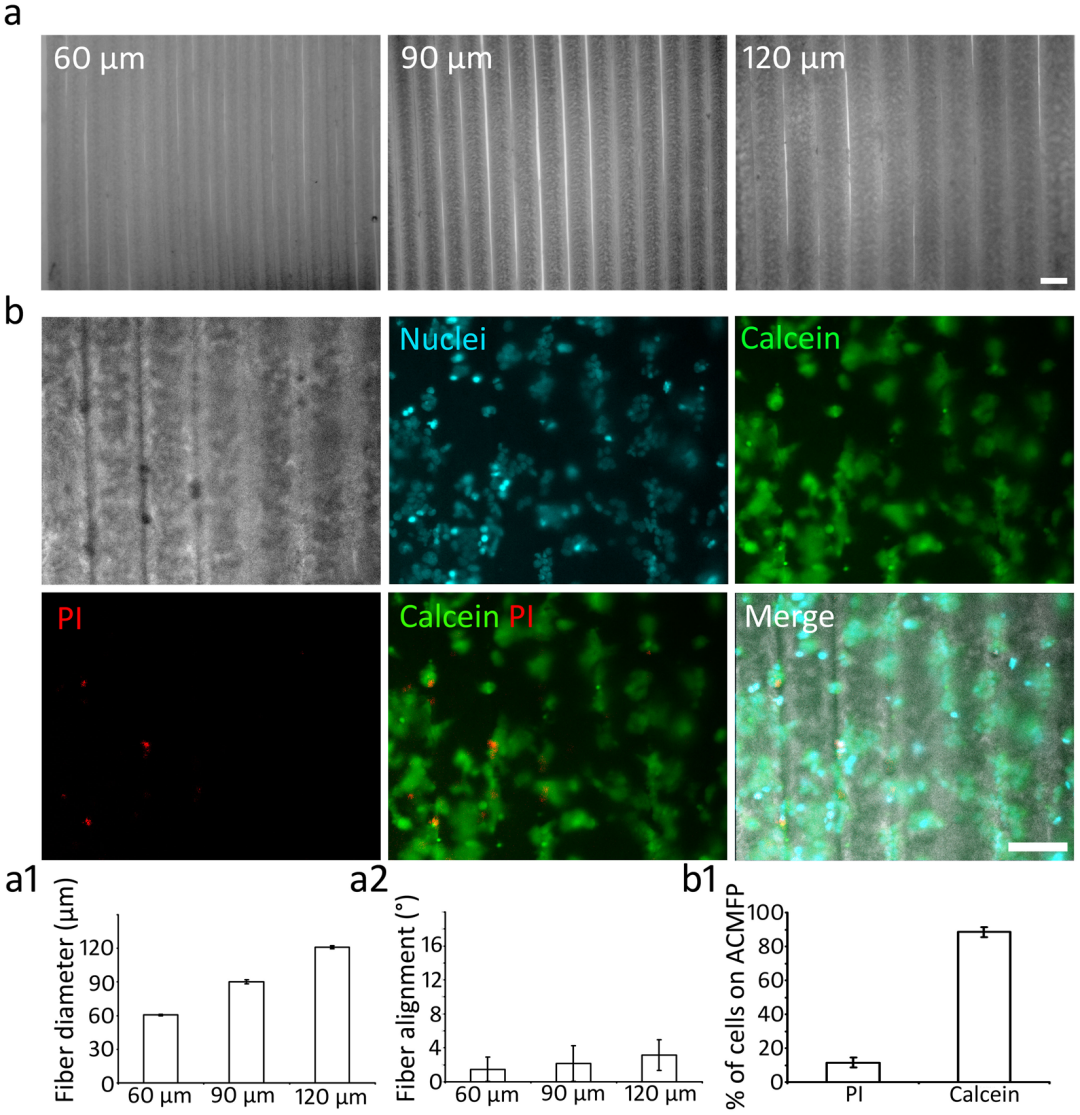
Fabrication of ACMFPs and evaluation of their diameter, angle, and biocompatibility. (**a**) Phase contrast images show 60, 90 and 120 μm diameter ACMFPs. (**b**) HEK293 cells cultured on the 90 μm ACMFP for 48 h. H33342 (Cyan, a fluorescent dye for nuclei staining), Calcein (Green, a marker for the live cell) and PI (Red, a marker for the dead cell) staining shows cell viability on ACMFP. (**a1**) The diameter of 60, 90 and 120 μm ACMFPs are measured by the ImageJ software (mean ± standard deviation shown in the figure; n = 18 samples per group). (**a2**) The alignment value of 60, 90, and 120 μm ACMFP is tested by the ImageJ software (mean ± standard deviation shown in the figure; n = 18 samples per group). (**b1**) HEK293 cells are labeled by H33342, Calcein, and PI after culture on the 90 μm ACMFP for 48 h. The quantitative study shows the viability of HEK293 on fibers (mean ± standard deviation shown in the figure; n = 6 samples per group). Scar bar: 100 μm.

It was observed that the ACMFP fiber diameter was related to the PLGA and glycerin micro-fluid flow speed. In this research, we aimed to test 3 different fiber diameters including 60 µm, 90 μm and 120 μm (Fig. 3a). When the pump speed of PLGA solution was set at 15 μl/min and glycerin solution at 500 μl/min, fibers of 60.61 ± 0.65 μm diameter were produced. The 90 μm diameter fibers (90.23 ± 1.75 μm) were obtained with PLGA solution at 20 μl/min and glycerin solution at 500 μl/min. The 120 µm (121.05 ± 1.23 µm) fibers were produced with PLGA solution at 28 μl/min and glycerin solution at 500 μl/min (mean ± standard deviation; Fig. 3a1). The ACMFP fibers also demonstrated high alignment with the fiber alignment values of 1.47 ± 1.43°, 2.15 ± 2.10° and 3.15 ± 1.79° for the 60, 90 and 120 μm fiber groups respectively (mean ± standard deviation; Fig. 3a2). Fibers with angles smaller than 5° were considered as aligned fiber bundles in this research.

The biocompatibility of PLGA has been demonstrated previously^22,23^. However, PLGA was treated with Pluronic F127 in this study. It is unclear whether F127-treated PLGA has any cytotoxicity. Therefore, its biocompatibility needs to be studied using a widely-used mammalian cell line such as the human cell line HEK293 cells. To test the biocompatibility of fibers, HEK293 cells were seeded on 90 µm ACMFP for 2 days (Fig. 3b). In H33342 (cell dye for fluorescent staining of DNA and nuclei), Calcein (labeling live cells) and Propidium iodide (PI; labeling dead cells) staining, 88.46 ± 2.81% cells on the fiber were Calcein positive, suggesting they were live cells. In contrast, 11.54 ± 2.81% cells were labeled by PI, which indicates dead cells (mean ± standard deviation; Fig. 3b1). The result indicates that the majority of HEK293 cells survived well on the fiber substrates, suggesting the biocompatibility of the ACMFP fiber system.

### Differentiation of ES cells on the ACMFP

**Figure 4:**
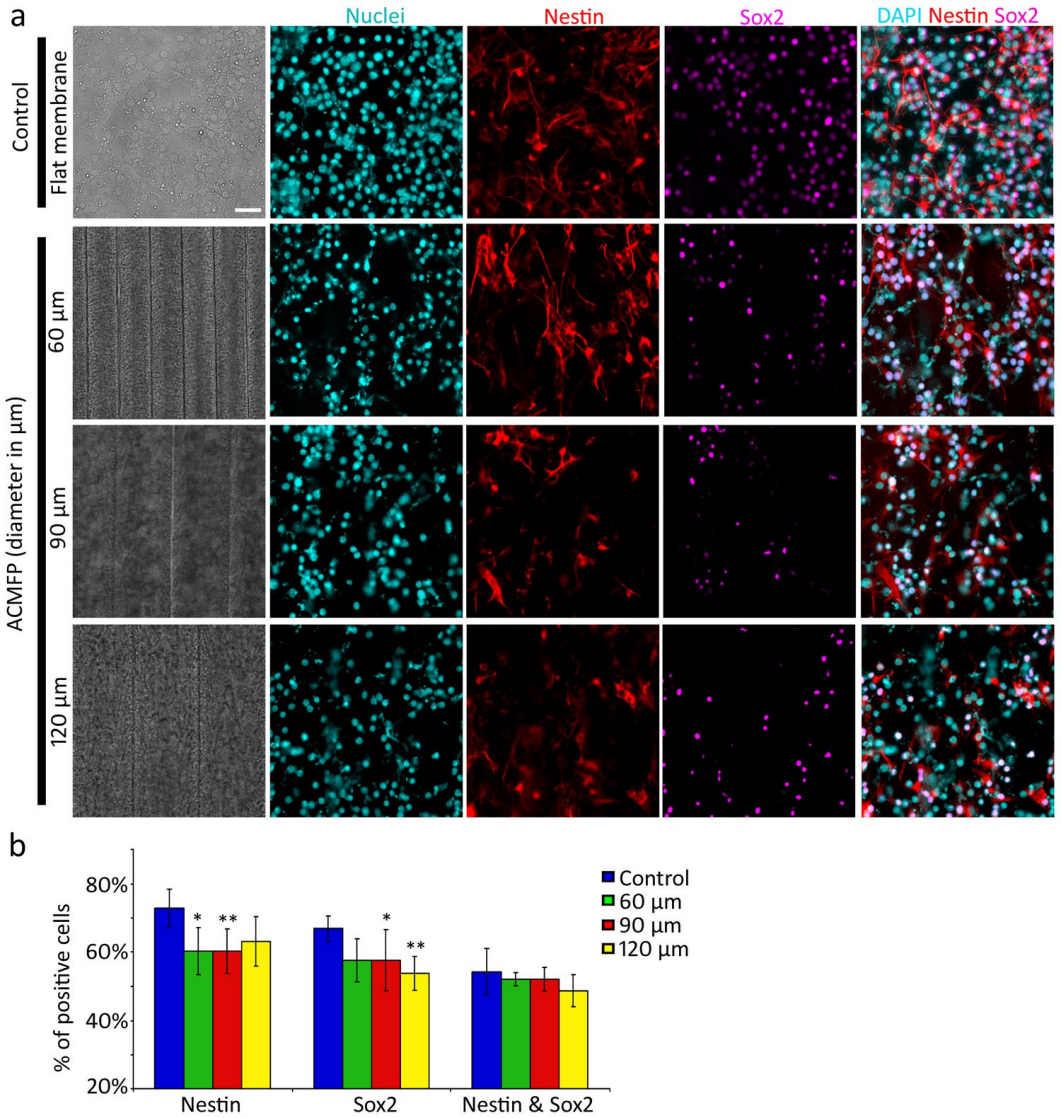
Evaluation of neural stem cell generation in the ACMFP and control groups. (**a**) Immunofluorescence displays nuclei (Cyan), Sox2 (Magenta) and Nestin (Red) staining for 60, 90 and 120 μm ACMFP as well as flat PLGA membrane control. (**b**) Quantification of the percentage of Nestin-positive cells, Sox2 positive cells and double-labeled cells in 60, 90, 120 μm ACMFP and flat PLGA membrane control groups (mean ± standard deviation shown in the figure, n = 6; **indicates P < 0.01; ANOVA followed by Tukey post hoc test, analysis results shown in Supplemental Table S2). (**c**) Quantification study of the number of Nestin-positive cells, Sox2 positive cells and double-labeled cells in 60, 90, 120 μm ACMFP and control (flat PLGA membrane) groups (mean ± standard deviation shown in the figure, n = 6 samples per group; **indicates P < 0.01; ANOVA followed by Tukey post hoc test, analysis results shown in Supplemental Table S2). Scar bar: 50 μm.

All-trans retinoic acid was used to guide CE1 mouse ES cells (CE-1 SCRC-1038 cell line; from American Type Culture Collection, ATCC) to differentiate into neural stem cells (NSCs) in this research^35–37^. Retinoic acid-treated CE1 cells were seeded on 60, 90 and 120 µm ACMFP as well as flat PLGA membrane (control) for 6 days. Immunostaining of the NSC markers Sox2 and Nestin was explored and the results showed the percentage of Sox2 and Nestin double-labeled cells as 44.23 ± 5.60%, 42.07 ± 1.92%, 39.73 ± 3.38% and 38.75 ± 4.69% in the control (flat PLGA membrane), 60, 90 and 120 µm ACMFP groups respectively (mean ± standard deviation; Fig. 4a,b). There was no significant difference between the ACMFP and control groups (P > 0.05, ANOVA-Tukey test; Supplemental Table S2). This study suggests that approximately 30–50% of ES cells become NSCs on ACMFP and the ACMFP may not influence NSC differentiation.

**Figure 5:**
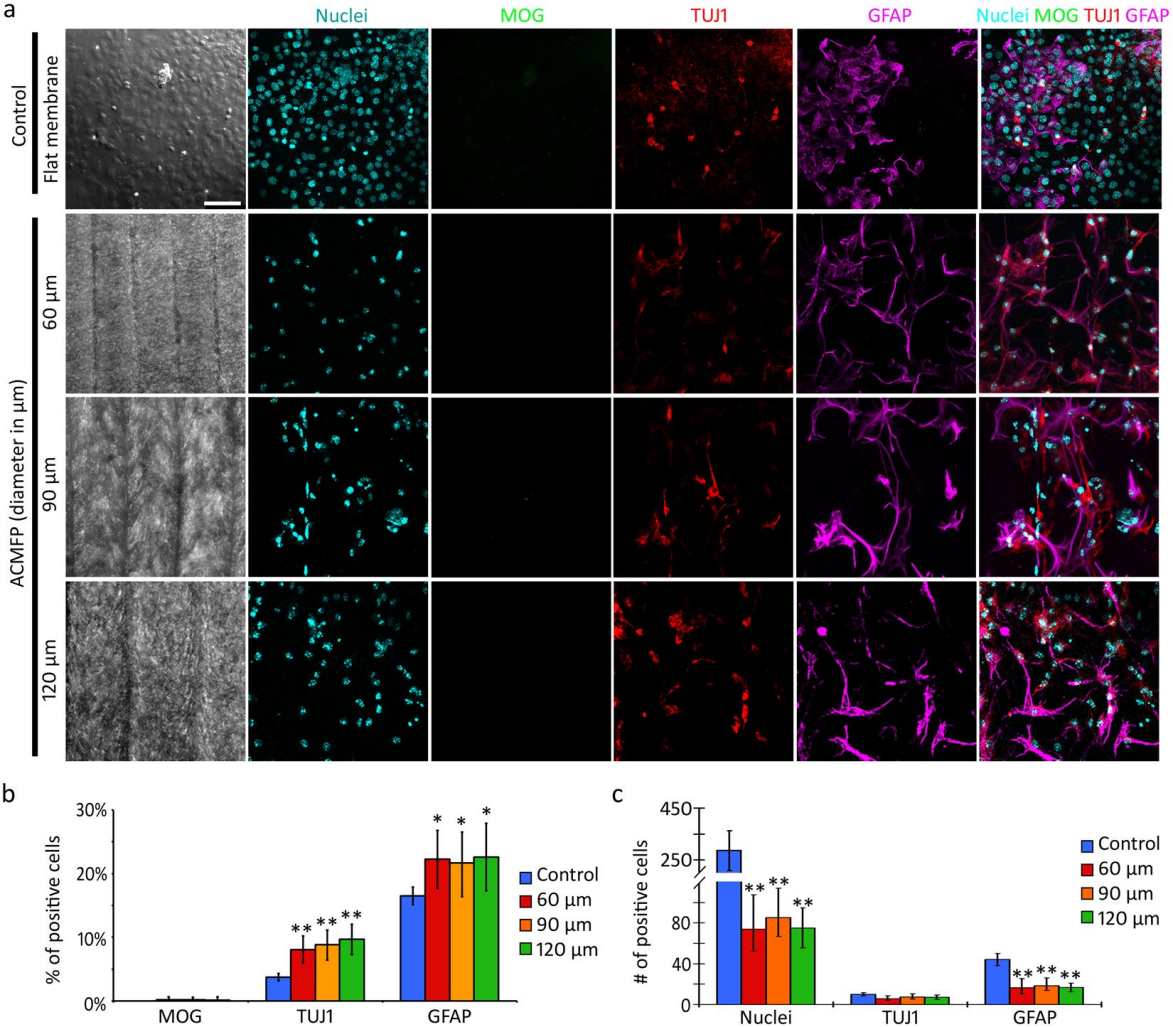
Neural differentiation of ES cells in the ACMFP and control groups. (**a**) Confocal-based DIC microscopy shows control (flat PLGA membrane), 60, 90 and 120 µm ACMFPs. Immunofluorescence and confocal microscopy demonstrate staining of Nuclei (Cyan), GFAP (Magenta, a marker for astrocyte cell), TUJ1 (Red, marker for neuron) and MOG (Green, a marker for oligodendroglia cell) in the 60, 90, 120 μm ACMFP and control (flat PLGA membrane) groups. (**b**) Quantification study of the percentage of MOG positive cells, TUJ1 positive cells and GFAP positive cells in 60, 90, 120 μm ACMFP and control (flat PLGA membrane) groups (mean ± standard deviation shown in the figure, n = 12 samples per group; *indicates P < 0.05; **indicates P < 0.01; ANOVA followed by Tukey post hoc test, analysis results shown in Supplemental Tables S3 and S4). (**c**) Quantification study of the number of MOG positive cells, TUJ1 positive cells and GFAP positive cells in 60, 90, 120 μm ACMFP and control (flat PLGA membrane) groups (mean ± standard deviation shown in the figure, n = 12 samples per group; *indicates P < 0.05; **indicates P < 0.01; ANOVA followed by Tukey post hoc test, analysis results shown in Supplemental Tables S3 and S4). Scale bar: 50 μm.

Neural cells usually include neurons (expressing neuron-specific class III β-tubulin; TUJ1), glial cells (expressing glial fibrillary acidic protein; GFAP) and oligodendrocytes (expressing myelin oligodendrocyte glycoprotein; MOG). To determine the neural differentiation of ES cells in the control (flat PLGA membrane) and ACMFP groups, neuronal (anti-TUJ1), oligodendrocyte (anti-MOG) and glial cell (anti-GFAP) specific antibodies were used (Fig. 5a). Immunofluorescence showed that the percentages of TUJ1 positive cells were 3.74 ± 0.59%, 8.08 ± 2.13%, 8.82 ± 2.29% and 9.67 ± 2.38% in the control (flat PLGA membrane), 60, 90 and 120 µm ACMFP groups respectively (mean ± standard deviation). Statistical analysis suggests that the percentage of TUJ1 positive cells of all 3 fiber groups was significantly higher than that of the control group (P < 0.01, ANOVA-Tukey test; Supplemental Table S3). There was no significant difference of TUJ1-expressing cells among 3 ACMFP groups (P > 0.05, ANOVA-Tukey test; Fig. 5b, Supplemental Table S4). The percentages of GFAP positive cells were 16.52 ± 1.38%, 22.26 ± 4.56%, 21.69 ± 4.86% and 22.58 ± 5.31% in the control, 60, 90 and 120 µm ACMFP groups respectively, which was significantly different (mean ± standard deviation; P < 0.05, ANOVA-Tukey test; Supplemental Table S3), whereas no significant difference was observed among 3 ACMFP groups (P > 0.05, ANOVA-Tukey test; Fig. 5b, Supplemental Table S4). Very few MOG positive cells were found in all groups (Fig. 5a), which excluded the possibility of analysis. These results suggest that the ACMFP stimulates the generation of neuronal and glia cells.

### Mechanism of increased neuronal differentiation on the ACMFP

We further analyzed the absolute number of TUJ1 and GFAP positive cells. After calibration of the curved surface of the ACMFP group, the number of TUJ1 positive cells was 9.91 ± 1.55, 5.77 ± 1.65, 7.36 ± 2.03 and 7.155 ± 2.22 per 0.3 mm^2^ in the control (flat PLGA membrane), 60, 90 and 120 µm groups respectively (mean ± standard deviation; 0.3^2^ mm is the area of an image captured by a 20 × objective), which was not significantly different (P > 0.05, ANOVA-Tukey test; Supplemental Tables S3 and S4). After calibration of the curved surface of the ACMFP group, the number of GFAP-expressing cells in the control group was significantly higher than all 3 ACMFP groups, 44.08 ± 5.93, 16.324 ± 5.89, 18.49 ± 4.75, and 16.589 ± 4.22 per 0.3 mm^2^ in the control, 60, 90 and 120 µm ACMFP groups respectively (mean ± standard deviation; P < 0.05, ANOVA-Tukey test; Supplemental Table S3). The number of GFAP-expressing cells was not significantly different among 3 ACMFP groups (P > 0.05, ANOVA-Tukey test, Supplemental Table S4). 4′,6-diamidino-2-phenylindole (DAPI) was used to count all nuclei in this research, which showed that the number of DAPI positive cells in the control group was 2-fold higher than all ACMFP fiber groups (P < 0.01, ANOVA-Tukey test; Fig. 5c; Supplemental Table S3).

**Figure 6:**
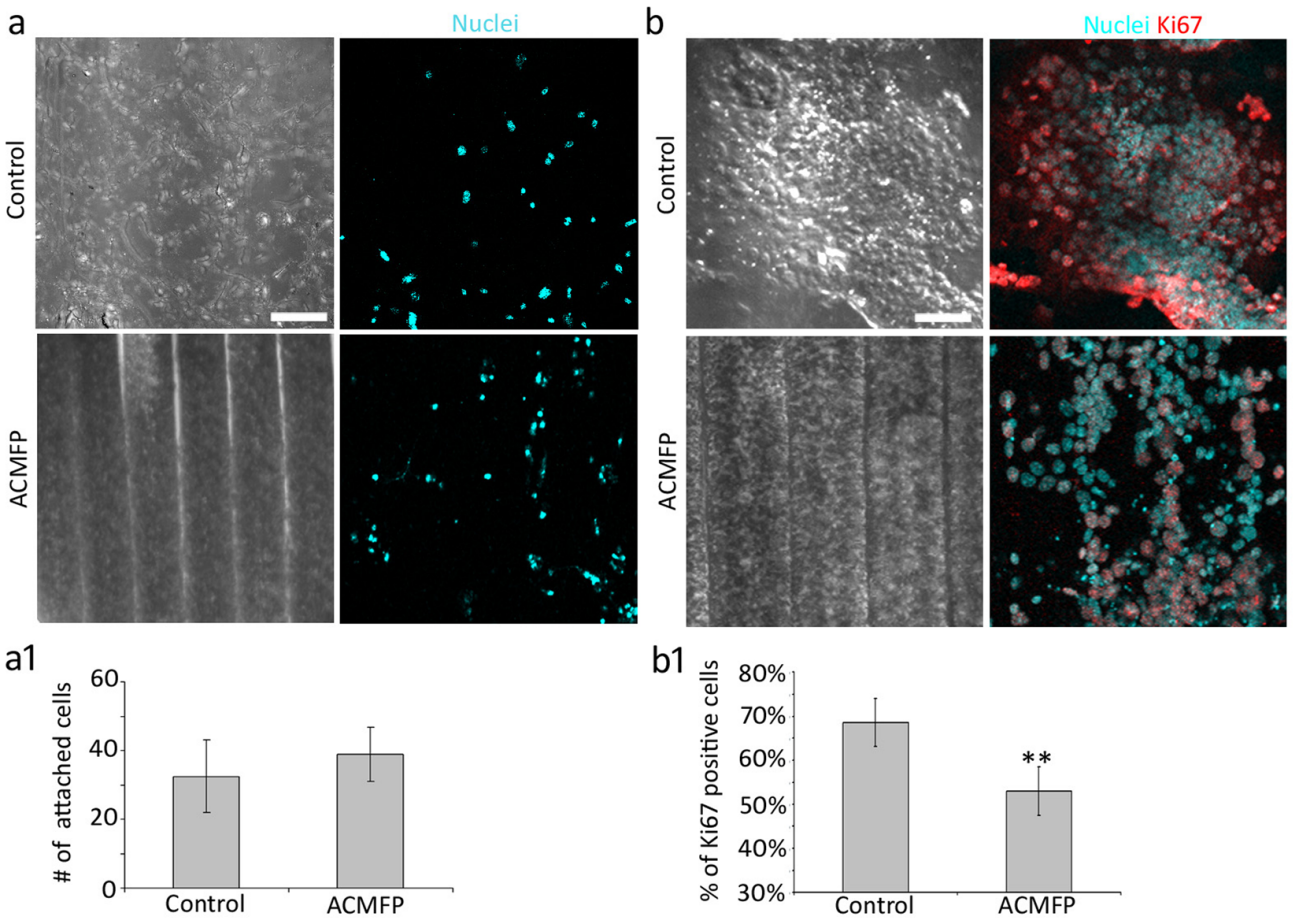
Evaluation of ES cell initial attachment and proliferation in the ACMFP and control (flat PLGA membrane) groups. (**a**) Retinoic acid-treated CE1 cells were cultured on the control and 90 μm ACMFP for 4 h and stained with nuclei dye H33342 (Cyan; confocal with DIC microscopy). (**a1**) Quantification of the number of total cells in the 90 μm ACMFP and control groups (mean ± standard deviation shown in the figure, n = 8 samples per group; *indicates P < 0.05; Student’s t-test, analysis results shown in Supplemental Table S5). (**b**) Confocal images show DAPI (Cyan) and Ki67 (Red, a marker for proliferation cell) staining of retinoic acid-treated CE1 cells on the control and ACMFP (confocal with DIC microscopy). (**b1**) Quantification of the percentage of Ki67 positive cells in the 90 μm ACMFP and control groups (mean ± standard deviation shown in the figure, n = 8 samples per group; *indicates P < 0.05; **indicates P < 0.01; Student’s t-test, analysis results shown in Supplemental Table S5). Scale bar: 100 μm in a and 50 μm in b.

We next examined two possibilities that may be related to increased neuronal differentiation and reduced total cell number in the ACMFP group: (a) less cell attachment and (b) limited cell proliferation in the ACMFP group. To study cell attachment, the same number of retinoic acid-treated CE1 cells were seeded to the 90 µm ACMFP and control (flat PLGA membrane) group. After 4 h when all cells had attached, H33342 was added to both groups (Fig. 6a). After calibration of the curved surface of the ACMFP group, the number of attached cells in the ACMFP group was 38.87 ± 2.67 per 0.3 mm^2^, whereas it was 32.47 ± 7.84 per 0.3 mm^2^ in the control group (mean ± standard deviation; Fig. 6a1; 0.3 mm^2^ is the area of an image captured by a 20 × objective). Statistical analysis did not show a significant difference (P > 0.05, Student’s t-test). It is observed that the cell number of the ACMFP group at 4 hr was 38.87 ± 2.67 whereas it was 85.35 ± 18.36 after 6 days, suggesting that the total cell number of the ACMFP group increased. Cell counting of the control group showed 32.47 ± 7.84 and 267.92 ± 38.22 cells at 4 hr and 6 days respectively. To further characterize cell proliferation, anti-Ki67 immunostaining was applied to both groups (Fig. 6b). The percentage of Ki67 positive cell was 68.52 ± 5.46% in the control group, whereas it was 53.00 ± 8.94% in the 90 µm ACMFP group, suggesting that remarkably more cells were in the proliferating stage in the control group (mean ± standard deviation; P < 0.01, Student’s t-test; Fig. 6b1, Supplemental Table S5). These results demonstrated that (a) the number of attached cells of the ACMFP group was similar to the control group, (b) the cell number of both control and ACMFP groups increased after 6 days, and (c) cell proliferation was significantly higher in the control group compared to the ACMFP group. This study suggests that a reduced cell proliferation in the ACMFP group may contribute to decreased total cell number of the ACMFP groups and increased neuronal differentiation rate of the ACMFP group.

### Neurite guidance on the ACMFP

**Figure 7:**
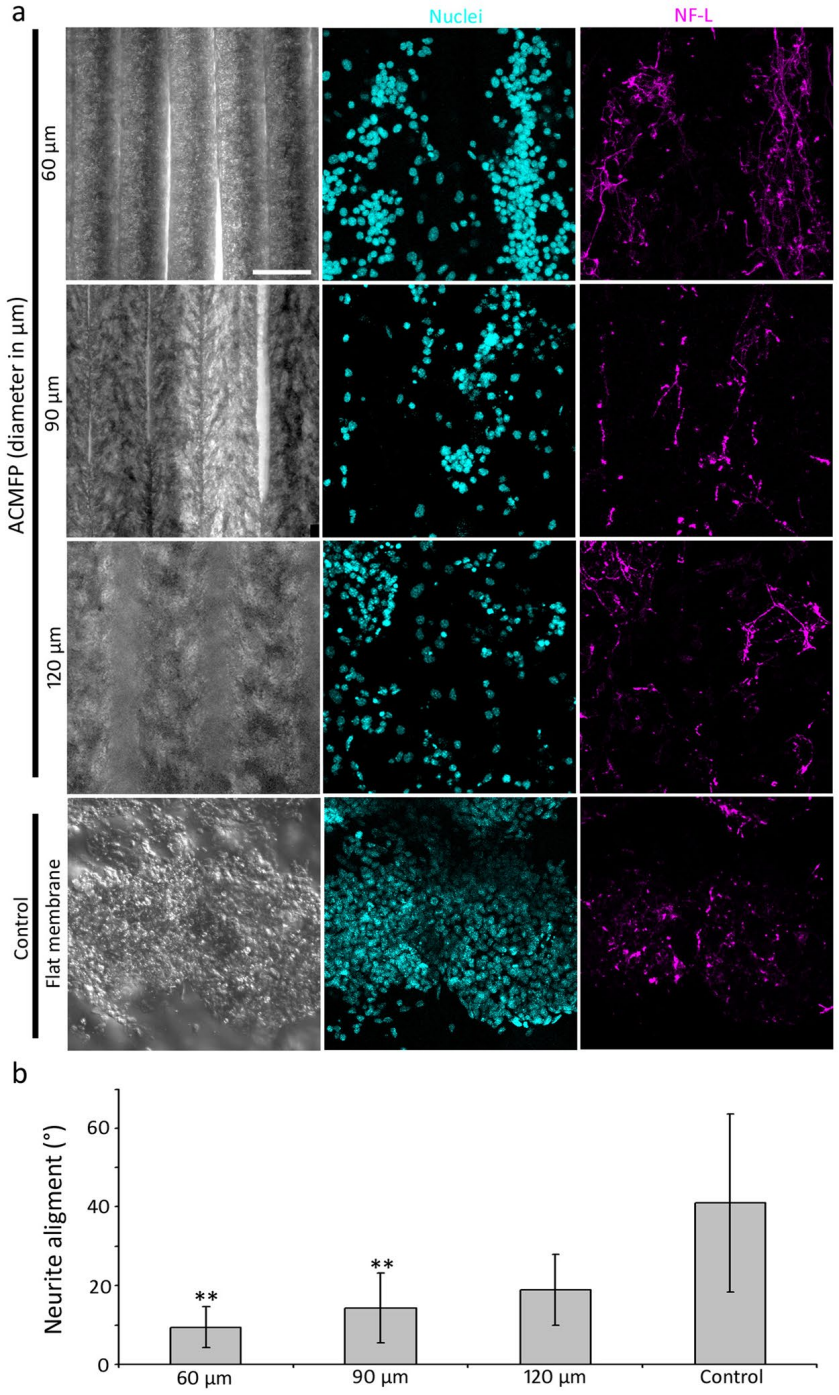
Neurite guidance in the 60, 90, 120 μm ACMFP and control (flat PLGA membrane) groups. (**a**) Confocal images show labeling of DAPI (Cyan), Neurofilament (NF-L; Magenta, marker for neurons and neurites) in the 60, 90, 120 μm ACMFPs and control groups (confocal with DIC microscopy). (**b**) Analysis of the neurite alignment in the 60, 90, 120 μm ACMFPs and control groups (mean ± standard deviation shown in the figure, n = 12 samples per group; *indicates P < 0.05; **indicates P < 0.01; ANOVA followed by Tukey post hoc test, analysis results shown in Supplemental Table S6). Scale bar: 100 μm.

Retinoic acid-treated CE1 mouse ES cells were seeded on the control (flat PLGA membrane), 60, 90 and 120 µm ACMFP and maintained in 37 °C incubator for 6 days, followed by anti-neurofilament immunostaining to examine neurite outgrowth directions. Neurite outgrowths were projected in a random direction in the control group (Fig. 7a). The alignment value of the control group ranged from 0° to 90°, with an average value of 41.07 ± 22.72° (mean ± standard deviation). A large standard deviation value suggests that the direction of neurites of the control group was random (Fig. 7b). In the ACMFP groups, neurite outgrowths were observed in a more parallel pattern. The average alignment values of 60, 90 and 120 µm ACMFP were 9.38 ± 5.18°, 14.27 ± 8.82° and 18.87 ± 9.02° respectively (mean ± standard deviation). Statistical analysis showed a significant difference of the alignment value in the control, 60, 90 and 120 µm ACMFP groups (P < 0.01, ANOVA-Tukey test; Supplemental Table S4). Tukey post hoc analysis demonstrated that the neurites of the 60 µm ACMFP group displayed the best alignment with a value lower than 10°, indicating that they were highly parallel. These results suggest that the diameter of ACMFP fiber affects the neurite outgrowth guidance and that the 60 µm ACMFP is an ideal substrate for neurite guidance.

## Discussion

Since the size of ES cells and their derivatives are usually in micrometers (~10 µm), it is generally accepted that only nano- or sub-micrometer biomaterials are able to affect ES cell differentiation into a neuronal lineage^12–15^. In contrast, micrometer biomaterial is hypothesized not capable of affecting cell fate determination of ES cells due to the relatively large size^14^. However, this hypothesis has not been thoroughly investigated. In this research, ACMFP was produced to test this hypothesis. After culturing of ES cells on the ACMFP and control (flat PLGA membrane) for 6 days, the percentage of cells expressing neuronal markers is significantly higher in the ACMFP groups, which suggests that ACMFP is able to stimulate neuronal differentiation of ES cells.

To understand the underlying mechanism of increased neuronal differentiation on ACMFP, a detailed cell counting and analysis were performed. It was observed at the end of the experiment that the absolute number of derived-neurons between the ACMFP and control groups is not significantly different; however, the total number of cells of the control group is approximately two times larger than the ACMFP group. It seems that the increased neuronal yield is likely due to lower total cell number after 6 days’ culture. The initial ES cell number in these two groups is identical, as the same number of ES cells were plated to the ACMFP and control groups. Two possibilities may explain the difference of final total cell numbers between two groups: (a) the cell attachment ability and (b) the cell proliferation capability. First, both ACMFP and control groups were coated with 0.1% gelatin and attached cell numbers were counted 4 hr after cell plating. A similar number of cells were found to attach to the ACMFP and control groups, suggesting that cell attachment less likely causes the lower final total cell number of the ACMFP group. Second, the cell proliferation assay displayed that cell proliferation is much slower in the ACMFP group, indicating that it may account for the lower final total cell number. Taken together, these two experiments suggest that the aligned ACMFP possesses the features of reduced cell proliferation rate, which may be related to the stimulation of neuronal differentiation.

Microfiber can influence the morphology and growth direction of a variety of cell types including neurons^15,34^. In this research, neurite outgrowth assay was evaluated in the ACMFP and control groups. It was found that the neurite alignment of the control group is very poor approximately 41° (0°−90°), which was randomly growing of neurites. In contrast, the alignment of neurite outgrowth of the ACMFP groups is much better: 9.38 ± 5.18°, 14.27 ± 8.82° and 18.87 ± 9.02° for 60, 90 and 120 µm diameter respectively. It is observed that neurites of the 60 µm group show the best alignment <10% (regarded as aligned outgrowth), whereas larger diameter ACMFP groups exhibit a less parallel pattern. This study indicates that the neurite outgrowth alignment is directly related to the diameter of ACMFPs, which is consistent with previous reports^15,34^ and may be used as an indicator for the design of future nerve repair research.

This research also includes several technical innovations and/or improvements. First, this research addresses how to fabricate ACMFP. In previous microfiber experiments, micrometer fiber production is usually not aligned and/or with limited alignment^12^. Since aligned fibers are critical for neurite outgrowth guidance, we have developed a novel fiber collection system, consisting of a syringe pump with a receiving motor system (Fig. 1). By the application of this fiber collection system, we are able to collect aligned ACMFPs (Fig. 1c,d). Second, this research solves the ACMFP shrinkage issue. In previous reports, micro-, submicron-, and nano-fibers are produced at room temperature; however, these fibers usually shrink significantly when they are exposed to 37 °C. Fiber shrinkage not only affects the alignment pattern of fibers but also influences cell fate determination as well as neurite outgrowth^38^, which remarkably limits the efficiency and application of this biomaterial. One possible reason for fiber shrinkage is the residual stress at higher temperature^38^. A previous study demonstrated that Pluronic F127 was able to prevent shrinkage of sub-micron fibers (0.5–2 µm). However, it is unclear whether Pluronic F127 is effective in preventing shrinkage of larger diameter fibers, as larger diameter fibers displayed a severely disorganized pattern. ACMFP shrinkage was observed in this research, and the fiber shrinkage as well as disorganization of 90- and 120-µm fibers was more severe than that of 60-µm fibers. We found that the application of Pluronic F127 is able to prevent fiber shrinkage for the 90-µm group, indicating that the anti-shrinkage effect of Pluronic F127 applies to large diameter ACMFPs.

In this report, an aligned ACMFP fabrication system was designed and established to study the neuronal differentiation/neurite outgrowth of ES cells. ACMFP fibers demonstrate the properties of reduced cell proliferation, increased neuronal differentiation and aligned neurite outgrowths of ES cell-derived neurons. The ACMFP system may provide a new model for the development of a multidisciplinary stem cell-based treatment option for nerve injuries.

## Methods

### ACMFP platform and flat PLGA membrane (control) fabrication

The microfluidic-based fabrication apparatus and procedures of PLGA micro-level fiber generation have been previously reported^34^, in which two syringe pumps were used and the fibers were collected by rotary coverslips. In our modified micro-fluidic fabrication apparatus, a third syringe pump was linked to the rotary coverslip, which was able to move the coverslips during fiber collection to prevent fiber overlapping (Fig. 1b,c). By changing the speed of the third pump and applying slight pressure with tweezers, the gap between microfibers was eliminated. Therefore, the new modified micro-fluidic fabrication apparatus included two parts. The first was the fabrication part which included two syringe pumps and polydimethylsiloxane (PDMS) microfluidic chip (Fig. 1c). Two syringe pumps separately pumped the core solution (10% PLGA 75:25 in dimethyl sulfoxide) and sheath solution (50% glycerol in distilled water; all from Sigma). Micro-level fibers were produced when two solutions met in the PDMS microfluidic chip. The size of fiber was controlled by the flow rate of two solutions. The second part was the receiving part which included a rotary coverslip (22 mm × 4 mm) attached to a pump driven syringe. The syringe-pump could move the rotary coverslip at a constant speed to prevent fiber overlapping (Fig. 1a,c). The core solution pumped at 15–30 µl/min with the sheath solution at 500 µl/min were tested to obtain fibers at a range of diameters of 30–150 µm (Data not show). The rotation speed of rotary coverslip was tested at 60 rpm in 40–200 µl/min to prevent fibers overlapping and make a limited gap microfiber pattern. ACMFP fibers were treated with distilled water for 3 h.

To generate a flat PLGA membrane as control, a custom-made glass chamber was produced on a glass slide surrounded by 0.17 µm thick coverslip (Supplemental Fig. S1b). 10% PLGA was poured into the chamber and dried at room temperature overnight, followed by a rinse with distilled water for 3 h. The PLGA membrane was peeled off from the glass slide by a tweezer to generate a flat PLGA surface as the control of ACMFP.

### Microfiber platform treatment

In Pluronic F127 treatment, flat PLGA membrane and ACMFP PLGA platforms were kept in 40% (m/v) Pluronic F127 solution (Sigma) at 4 °C overnight, 70 °C for 3 hr, followed by an ice-cold distilled water rinse for 3–5 times to remove residual Pluronic F127. Before cell seeding, samples were sterilized with 70% alcohol for 15 min, rinsed by PBS for 3–5 times, coated with 0.1% gelatin and placed at 37 °C for a minimum of 30 minutes for future use.

### Microfiber diameter and alignment test

Different diameter fibers were imaged by light microscopy (Leica DMI 3000B). The linear measurement tool from the ImageJ software diameter (National Institute of Health, NIH, USA) was used to measure fibers (n = 18 samples, 1–3 images were randomly selected per sample). The same image was used to measure the fiber alignment by the ImageJ software. One fiber was randomly chosen as a baseline reference annotated as 0°. The alignment value was determined by the angle between the reference fiber and other fibers using the angle measurement tool (n = 18 samples, 1–3 images were randomly selected per sample).

### Microfiber platform biocompatibility evaluation

Gelatin coated 90 μm ACMFP fibers were incubated in DMEM high-glucose (Sigma), 10% FBS (Atlanta) and 0.1% Ampicillin (Invitrogen) for 30 min in a 37 °C incubator supplies with 5% CO_2_. HEK293 cells were seeded on fibers and maintained in the incubator. After 24 hr, samples were stained with Calcein (1:2000), Propidium iodide (PI; 1:2000) and H33342 (1:3000; all from Invitrogen) for 20 min in the incubator, followed by imaging with Leica DMI 3000B epifluorescence microscopy.

### ES cell culture on ACMFP or flat PLGA membrane (control)

An embryonic stem (ES) cell line CE1, purchased from American Type Culture Collection (ATCC), was cultured in DMEM/F12 GlutaMAX with the supplementation of 10% knockout fetal bovine serum (FBS; Invitrogen), 1% non-essential amino acid (NEAA), 1% penicillin-streptomycin (P/S), 0.1% 2-mercaptoethanol (all from Invitrogen) and 1000 unit/ml leukemia inhibitory factor (LIF; Millipore) in the incubator. CE1 cells were dissociated with TryplE (Invitrogen) and cultured in LIF-free ES culture medium for 10 h. All-trans retinoic acid (10^−7^ M, Sigma) was added to the culture medium. Six days later, retinoic acid-treated CE1 cells were treated with TryplE (Invitrogen) for 3 min and dissociated cells were seeded on ACMFP (60, 90 and 120 μm) or flat PLGA membrane (control) and maintained in DMEM/F12 GlutaMAX, 10% FBS, 1% NEAA, 1% P/S, 0.1% 2-mercaptoethanol and 10 ng/ml nerve growth factor (Invitrogen) for 6 days.

### Neural differentiation of ES cells on the ACMFP

To characterize differentiation, retinoic acid-treated CE1 cells were cultured on either ACMFP fibers or flat PLGA membrane (control) for 6 days as described above. Cells were fixed in 4% paraformaldehyde, blocked with donkey serum in 0.5% Triton X-100 for 30 min, incubated with primary antibodies at 4 °C overnight followed by secondary antibody treatment at room temperature for 2 hr. Primary antibodies used in this study included: Anti-Sox2 (1:200; Abcam), Anti-Nestin (1:200; Developmental Studies Hybridoma Bank), Anti-TUJ1 (1:500; AVES), Anti-GFAP (1:200; Santa Cruz Biotechnology) and Anti-MOG (1:200; Millipore) antibodies. Secondary antibodies included Dylight 549 or Dylight 649 conjugated donkey anti-mouse, rabbit or chicken antibodies (Jackson Immunoresearch). 4,6-Diamidino-2-Phenylindole (DAPI; 1:500; Invitrogen) was used to label all nuclei. Samples were observed and imaged by Leica SPE confocal microscopy and/or Leica DMI 3000B epifluorescence microscopy. Cells cultured on coverslips serve as controls. The number of Sox2 and Nestin-positive cells, TUJ1 positive cells, GFAP positive cells and MOG positive cells were quantified via the Cell Counter plugin of the ImageJ software), and (Sox2 and Nestin double-labeled cells)/(DAPI positive cells) × 100% were calculated (n = 12 samples per group, 1–2 images were randomly selected per sample).

### Cell attachment and proliferation evaluation on ACMFP

To determine cell attachment, the same number of retinoic acid-treated CE1 cells were seeded on the 90 μm ACMFP microfibers or flat PLGA membrane (control) for 4 hr. The samples were stained with H33342 for 20 min to label nuclei and visualized via Leica SPE confocal microscopy. Nuclei were counted utilizing the Cell Counter plugin of ImageJ software (n = 8 samples per group, 1–2 images were randomly selected per sample).

To characterize cell proliferation, retinoic acid-treated CE1 spheres were cultured on ACMFPs or control flat PLGA membrane and fixed as above. Anti-Ki67 (1:200; ThermoFisher) with Dylight 549 conjugated donkey anti-rabbit secondary antibodies were used to label proliferating cells. The samples were observed and imaged by Leica SPE confocal microscopy. The number of Ki67 positive cells was counted as above, and (Ki67 positive cells)/(DAPI positive cells) × 100% was calculated (n = 8 samples per group, 1–2 images were randomly selected per sample).

### Neurites outgrowth evaluation on ACMFP

To characterize the alignment of neurite outgrowths, retinoic acid-treated CE1 cells were cultured on fibers or control PLGA membrane and fixed as above. Anti-neurofilament (1:200; Santa Cruz Biotechnology) with Dylight 649 conjugated donkey anti-mouse secondary antibodies were used to define neurites outgrowth. The samples were observed and imaged by Leica SPE confocal microscopy. The alignment value was used to determine the alignment of neurite outgrowth, which was determined by the angle between the neurite outgrowth (neurofilament positive neurites) and the microfibers. The linear measurement tool of the ImageJ software was used to determine the alignment value of neurofilament-positive cells (n = 12 samples per group, 8–10 neurons were randomly selected per sample; Supplemental Fig. S1a).

### Statistical analysis

All data were collected from at least 3 independent experiments (n = 6–18 samples per group) and presented as a mean ± standard deviation. Student’s t-test and ANOVA followed with Tukey test were used for statistical significance.

### Data availability

All data generated or analyzed in this study are included in this published article (and its Supplementary Information files).

## Electronic supplementary material

supplementary info
