# Supplementary material for: Aligned contiguous microfiber platform enhances neural differentiation of embryonic stem cells
Source: Sci Rep. 2018 Apr 17;8:6087. doi: 10.1038/s41598-018-24522-9 (PMC5904125; doi:10.1038/s41598-018-24522-9)
Supplement: Supplementary file 1 — supplementary info [file 41598_2018_24522_MOESM1_ESM.pdf]

Aligned contiguous microfiber platform enhances neural differentiation of embryonic stem cells

Zhenjie Liu, Zhengqing Hu*

Department of Otolaryngology-HNS

Wayne State University School of Medicine

Detroit, Michigan 48201, USA

*Corresponding Author:

Zhengqing Hu, MD, PhD

Department of Otolaryngology-HNS

Wayne State University School of Medicine,

550 E Canfield Street, 258 Lande

Detroit, MI 48201, USA

Phone: 313-577-0675

Fax: 313-577-8555

Email: zh@med.wayne.edu

Supplemental data and legend

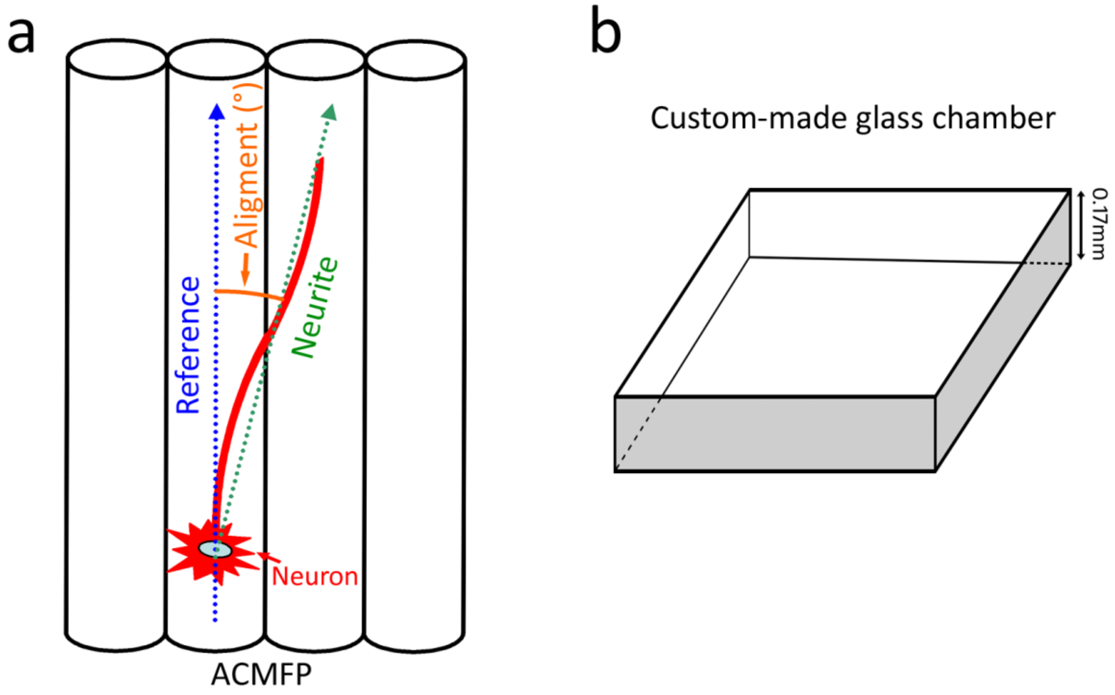

Figure S1. Methods for neurite alignment calculation and custom-made glass chamber for flat PLGA membrane fabrication.

(a) ACMFP direction is chosen as reference. Neurite alignment (orange) is equal to the angle between reference (blue) and neurite direction (green).

(b) Diagram for custom-made glass chamber. The thickness of chamber is 0.17 mm.

Table S1. ANOVA analysis of Fig. 2 (control and Pluronic F127 groups)

Fiber diameter of the control (PBS) group

Source of Variation	***SS***	***df***	***MS***	***F***	***P-value***
Treatment	16550.95	6	2758.49	230.5293	1.11e-16
Error	753.8523	63	11.9659		
Total	17304.81	69			

Fiber alignment of the control (PBS) group

Source of Variation	***SS***	***df***	***MS***	***F***	***P-value***
Treatment	862.2805	6	143.7134	9.9953	9.79e-08
Error	905.8235	63	14.3782		
Total	1768.10	69			

Fiber diameter of the Pluronic F127 group

Source of Variation	***SS***	***df***	***MS***	***F***	***P-value***
Treatment	93.5058	5	18.7012	1.4987	0.2056
Error	673.8269	54	12.4783		
Total	767.3327	59			

Fiber alignment of the Pluronic F127 group

Source of Variation	***SS***	***df***	***MS***	***F***	***P-value***
Treatment	14.4758	5	2.8952	2.2811	0.0593
Error	68.5379	54	1.2692		
Total	83.0136	59			

Table S2. ANOVA analysis of Fig. 4 (control, 60, 90 and 120 ACMFP groups)

% of Nestin positive cells

Source of Variation	***SS***	***df***	***MS***	***F***	***P-value***
Treatment	0.1091	3	0.0364	8.4041	0.0008
Error	0.0866	20	0.0043		
Total	0.1957	23			

% of Sox2 positive cells

Source of Variation	***SS***	***df***	***MS***	***F***	***P-value***
Treatment	0.0628	3	0.0209	5.2641	0.0077
Error	0.0796	20	0.004		
Total	0.1424	23			

% of Nestin & Sox2 double labeled cells

Source of Variation	***SS***	***df***	***MS***	***F***	***P-value***
Treatment	0.0109	3	0.0036	1.738	0.1915
Error	0.0417	20	0.0021		
Total	0.0525	23			

Table S3. ANOVA analysis of Fig. 5 (control, 60, 90 and 120 ACMFP groups)

% of TUJ1 positive cells

Source of Variation	*SS*	*df*	*MS*	*F*	*P-value*
Treatment	0.0251	3	0.084	20.6125	1.71e-18
Error	0.0179	44	0.0004		
Total	0.043	47			

% of GFAP positive cells

Source of Variation	*SS*	*df*	*MS*	*F*	*P-value*
Treatment	0.0293	3	0.0098	5.0936	0.0041
Error	0.0843	44	0.0019		
Total	0.1136	47			

Number of DAPI positive cells

Source of Variation	*SS*	*df*	*MS*	*F*	*P-value*
Treatment	438645.02	3	146215.01	122	1.11e-16
Error	52747.51	44	1198.81		
Total	491392.53	47			

Number of TUJ1 positive cells

Source of Variation	*SS*	*df*	*MS*	*F*	*P-value*
Treatment	22.7321	3	7.5774	1.876	0.1475
Error	177.7084	44	4.0388		
Total	200.4405	47			

Number of GFAP positive cells

Source of Variation	*SS*	*df*	*MS*	*F*	*P-value*
Treatment	8886.66	3	2962.22	57.959	2.55e-15
Error	2248.78	44	51.1086		
Total	11135.44	47			

Table S4. ANOVA analysis of Fig. 5 (60, 90 and 120 ACMFP groups)

% of TUJ1 positive cells among ACMFP groups

Source of Variation	*SS*	*df*	*MS*	*F*	*P-value*
Treatment	0.0015	2	0.0008	1.4378	0.2519
Error	0.0175	33	0.0005		
Total	0.019	35			

% of GFAP positive cells among ACMFP groups

Source of Variation	*SS*	*df*	*MS*	*F*	*P-value*
Treatment	0.0005	2	0.0002	0.0979	0.907
Error	0.082	33	0.0025		
Total	0.0825	35			

Number of DAPI positive cells among ACMFP groups

Source of Variation	*SS*	*df*	*MS*	*F*	*P-value*
Treatment	2313.39	2	1156.69	1.1452	0.3305
Error	33331.83	33	1010.06		
Total	35645.22	35			

Number of TUJ1 positive cells among ACMFP groups

Source of Variation	*SS*	*df*	*MS*	*F*	*P-value*
Treatment	44.2222	2	22.1111	2.224	0.1241
Error	328.0833	33	9.9419		
Total	372.3056	35			

Number of GFAP positive cells among ACMFP groups

Source of Variation	*SS*	*df*	*MS*	*F*	*P-value*
Treatment	83.3889	2	41.6944	0.6458	0.5307
Error	2130.50	33	64.5606		
Total	2213.89	35			

Table S5. Student's t-test analysis of Fig. 6 (control and 90 μm ACMFP groups)

Number of attached cells after 4 h culture

	Control	ACMFP
Mean	32.47	38.87
SD	7.84	2.86
SEM	2.77	1.01
n	8	8
T value	2.0012	
df	14	
P value	0.0651	

% of Ki67 positive cells after 6 days culture

	Control	ACMFP
Mean	0.6853	0.5300
SD	0.0546	0.0894
SEM	0.0193	0.0316
n	8	8
T value	4.1916	
df	14	
P value	0.0009	

Table S6. ANOVA analysis of Fig. 7 (control, 60, 90 and 120 ACMFP groups)

Neurite alignment

Source of Variation	***SS***	***df***	***MS***	***F***	***P-value***
Treatment	58751.36	3	19583.79	110.3932	1.11e-13
Error	70250.54	396	177.4004		
Total	129001.91	399			
